# Protocol for a mixed-methods and multi-site assessment of the implementation process and outcomes of a new community-based frailty programme

**DOI:** 10.1186/s12877-022-03254-6

**Published:** 2022-07-15

**Authors:** Woan Shin Tan, Ze Ling Nai, Hwee Teng Robyn Tan, Sean Nicholas, Robin Choo, Mimaika Luluina Ginting, Edward Tan, Poh Hoon June Teng, Wee Shiong Lim, Chek Hooi Wong, Yew Yoong Ding, the Geriatric Services Hub Programme Group, Santhosh Kumar Seetharaman, Christopher Tsung Chien Lien, Barbara Helen Rosario, Shou Lin Low, Arron Seng Hock Ang, Mei Foon Yap, Milawaty Nurjono, Lydia Au, Lian Leng Low, Su Fee Lim, Esther Li Ping Lim, Laura Bee Gek Tay, Germaine Hwui San Chng, Melvin Peng Wei Chua, Yee Sien Ng

**Affiliations:** 1 Geriatric Education & Research Institute, 2 Yishun Central 2, 768024 Singapore, Singapore; 2 Health Services & Outcomes Research Department, National Healthcare Group, Singapore, Singapore; 3 Social Service Research Centre, National University of Singapore, Singapore, Singapore; 4 Department of Geriatric Medicine, Institute of Geriatrics & Active Ageing, Tock Seng Hospital, Singapore, Singapore; 5 Tsao Foundation, Singapore, Singapore

**Keywords:** Frailty care, Comprehensive geriatric assessment, Care coordination, Multi-disciplinary team care

## Abstract

**Background:**

Frailty is increasing in prevalence internationally with population ageing. Frailty can be managed or even reversed through community-based interventions delivered by a multi-disciplinary team of professionals, but to varying degrees of success. However, many of these care models’ implementation insights are contextual and may not be applicable in different cultural contexts. The Geriatric Service Hub (GSH) is a novel frailty care model in Singapore that focuses on identifying and managing frailty in the community. It includes key components of frailty care such as comprehensive geriatric assessments, care coordination and the assembly of a multi-disciplinary team. This study aims to gain insights into the factors influencing the development and implementation of the GSH. We also aim to determine the programme’s effectiveness through patient-reported health-related outcomes. Finally, we will conduct a healthcare utilisation and cost analysis using a propensity score-matched comparator group.

**Methods:**

We will adopt a mixed-methods approach that includes a qualitative evaluation among key stakeholders and participants in the programme, through in-depth interviews and focus group discussions. The main topics covered include factors that affected the development and implementation of each programme, operations and other contextual factors that influenced implementation outcomes. The quantitative evaluation monitors each programme’s care process through quality indicators. It also includes a multiple-time point survey study to compare programme participants’ pre- and post- outcomes on patient engagement, healthcare services experiences, health status and quality of life, caregiver burden and societal costs. A retrospective cohort study will compare healthcare and cost utilisation between participants of the programme and a propensity score-matched comparator group.

**Discussion:**

The GSH sites share a common goal to increase the accessibility of essential services to frail older adults and provide comprehensive care. This evaluation study will provide invaluable insights into both the process and outcomes of the GSH and inform the design of similar programmes targeting frail older adults.

**Trial Registration:**

ClinicalTrials.gov Identifier NCT04866316. Date of Registration April 26, 2021. Retrospectively registered.

## Background

Frailty denotes a state of increased vulnerability due to an age-associated decline in function and reserve such that the ability to cope with day-to-day or acute stressors is compromised [1]. As a result, frail older adults are usually more susceptible to adverse outcomes, including disability, hospitalisation, and mortality [2]. A representative population study in the United States reported a frailty prevalence of 15% amongst community-dwelling older adults [3]. Meanwhile, a study of older adults by the United Kingdom (UK) Biobank reported 39% as pre-frail and 4% as frail [4]. Similar trends are observed in Singapore, an Asian island-state of 5.7 million [5] and one of the world’s most rapidly ageing countries [6]. The reported prevalence of frailty in Singapore ranged from 5.7 to 6.2% with a corresponding 37–46% for pre-frailty [7–9] among community-dwelling older adults, depending on the population studied and identification tool used [10].

Frailty can be managed and even reversed [11]. With almost 40% of our population assessed as frail or in danger of becoming frail, there is a strong need to equip the Singapore healthcare system to identify and manage frailty [12]. To accomplish this, whilst addressing the complex care needs of frail older adults, care has shifted from a disease-specific to a comprehensive approach [13]. Comprehensive care for frail older adults usually comprises several components, including comprehensive geriatric assessments (CGAs), multi-disciplinary teams, individualised care plans (ICPs) and a variety of services to cater to their healthcare and social needs [14]. Screening for frailty can identify individuals most likely to benefit from a CGA and targeted interventions [15]. In 2017, England became the first to mandate frailty assessment for adults aged 65 years and older. The widespread deployment of the electronic frailty index, which automatically grades frailty using data available in the primary care electronic medical records [16], supported this national effort.

Innovative care models involving a combination of the above components have emerged to provide more comprehensive care [17] and improve frail older adults’ health and social outcomes [18]. Greater integration of care among healthcare professionals of different disciplines and settings for frail older adults has reduced hospitalisations [19] and supported the maintenance of functional mobility over 12-months [20]. A systematic review of integrated or coordinated care found that multi-component care models were more likely to increase patient satisfaction, patients’ accessibility to and perceived quality of care [21]. However, there was inconsistent and limited evidence on how these care models influence healthcare costs and outcomes of frail older adults [21–23]. The evidence above suggests the need for more evidence to determine the effectiveness of comprehensive, integrated care models for frail older adults.

Integrated or coordinated care usually comprises partnerships or collaborations between at least two healthcare service providers, such as hospitals and primary care providers [24]. Partners in these care models collaborate by sharing expertise through training, whereby members of the geriatric expert teams train the primary care staff and nurses to build capabilities in comprehensive care of frail older adults [25, 26]. Other care models have also found that collaborators themselves conduct comprehensive healthcare and assessments to identify relevant health and social needs and follow up with patients through home visits [27] or by directing them to suitable care services [28]. Most care models also include shared discussions about the patients through multi-disciplinary team meetings. In a review of 28 integrated care programmes [29], provider commitment and trusting relationships were foundational to effective collaborations, communication and knowledge sharing among multi-disciplinary teams. Successful programme implementation was dependent on the quality of leadership and the leaders’ efforts to instil a shared vision and create an organisational culture that supports practice changes and joint governance.

This paper describes the evaluation protocol for a mixed-methods, multi-site evaluation of the Geriatric Services Hub (GSH), a programme for frail older adults in Singapore. The GSH is a novel intervention in the local context. It comprises core components of multi-component frailty care programmes, including CGAs and individually-tailored multi-factorial intervention delivered by a multi-disciplinary team [30]. The transferability of implementation insights and outcomes from prior frailty programmes, which are largely derived from Western studies, cannot be assumed, since the eventual results of implementation are dependent on the context and culture that the health system is situated within. Moreover, evidence on the outcomes of these intervention programmes is mixed [18, 23], with few insights regarding which aspects (programme logic or implementation process) need to be adjusted. Better understanding of the implementation context would support practitioners and policymakers to ensure complex interventions achieve their intended outcomes. Hence, the specificity of cultural context in the unique healthcare system of Singapore and the lack of understanding of stakeholder perceptions warrant a comprehensive evaluation of the GSH model. In the next segment, we will briefly describe the GSH programme and its position in Singapore’s healthcare system.

## Geriatric services hubs

The Singapore Healthcare System consists of three regional healthcare systems (RHS) in the central, eastern, and western regions of Singapore. These RHS were established to coordinate and organise healthcare service providers, integrate care across providers, and manage population health for their respective regions. Hence, each RHS consists of a network led by a major public hospital collaborating with other healthcare providers such as primary care, day rehabilitation, and community hospitals within the same geographical region [31]. Each RHS is provided with funding to implement programmes to deliver comprehensive care beyond the hospital to the community. However, fragmentation in healthcare delivery continues to exist between and within each RHS. There are other forms of commonly utilised care services that are privately owned and not under the jurisdiction of the RHS, which include daycare, day rehabilitation centres, private clinics and other variations of healthcare and allied health services. Therefore, although geographically located within the same region, differences in governance and financing structures between the RHS-funded services and privately-owned facilities result in insufficient information transfer, capabilities and capacities between healthcare providers, and acute hospital-centricity.

**Fig. 1:**
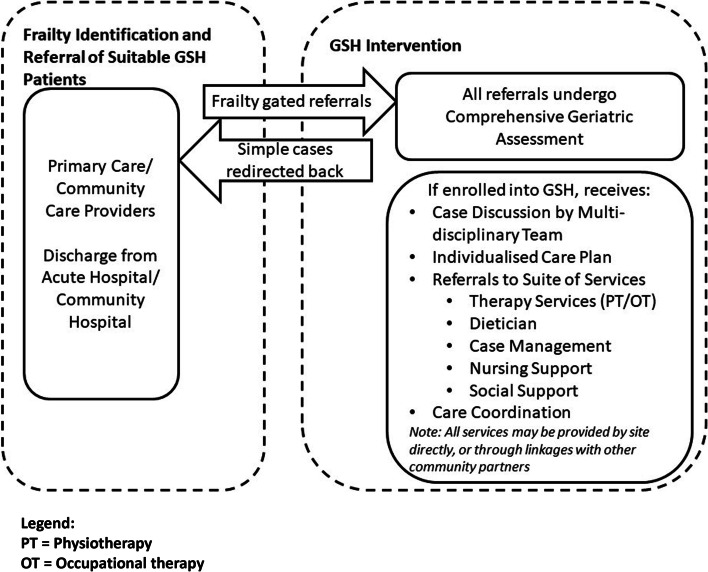
Functions of the Geriatric Services Hub Intervention

As part of a national effort to engage and support frail older adults in Singapore, community volunteers are trained to screen for frailty amongst older adults while community nurses are engaged to conduct rapid and targeted geriatric and frailty assessments at neighbour-based nursing posts [32]. Despite these ongoing efforts, the link between frailty assessments and the wider network of community care providers needs to be strengthened. To address this gap, the Singapore Ministry of Health provided funding to test a new programme for frail adults aged 65 years old and older – the GSH. The GSH is a multi-disciplinary care model that focuses on identifying and managing frailty in the community. It is currently piloted by 5 acute hospitals that partner with primary care providers, community health and social service providers and various hospital sites to deliver care for community-dwelling older adults. As shown in Fig. 1, the key activities involved are (1) frailty identification, (2) frailty assessment using a CGA, followed by the (3) conduct of multi-disciplinary meetings (MDMs) involving a multidisciplinary team comprising of geriatricians, geriatric nurses and allied healthcare professionals. As a result of the MDMs, (4) individualised care plans are developed and implemented, followed by (5) referrals to frailty-related services and (6) care coordination to facilitate the utilisation of such services. The Ministry of Health has specified the use of the Clinical Frailty Scale (CFS) [33] to assess frailty in the GSH and to support the enrollment of older adults living with very mild, mild, moderate or severe frailty into the GSH programme. This study protocol will focus on the evaluation of these five pilot sites. Due to geographical locations, the five pilot sites fall under the jurisdiction of two different RHS in Singapore. Each site focuses on its existing strengths and resources to ride on or build new partnerships within its RHS to provide community-anchored referral-gated geriatric care. Through these partnerships, the GSH functions as a network of providers led by a core team of acute hospital-based healthcare professionals [34]. Each site acts as a consolidation point in the community by actively receiving referrals from various service providers. All GSH sites share the following goals:

- To provide early identification of frailty and offer comprehensive and coordinated care in the community through collaborative partnerships with partners, including polyclinics, general practitioners (GPs), and community health and social service providers.
- To provide CGAs to identify needs of frail older adults and to establish a care plan for each individual patient.
- To increase frail older adults’ access to essential services and transition across shared primary care and other community-based providers more seamlessly.
- To provide core services of geriatric assessments, nursing support, therapy service and care coordination and/or case management.
- To build capabilities by providing training to the primary care staff in identifying, caring and managing frail older adults.

Despite these common goals, each GSH was designed differently to best harness their existing strengths and resources. As a result, each GSH site focuses on a different mode of operation. Table 1 provides an overview of the different programmes based on their funded components.

**Table 1:** Funded components in pilot care models in the five Geriatric Services Hub sites

Regional Health System	Study Site	Programme Description	Population Targeted	Primary Referral Source	Main Setting and delivery	Programme Lead(s)
National University Health System	Alexandra Hospital	Geriatrician assesses older adults for frailty and manages patients in the primary care setting	Age: 65 + CFS: 4–7	General Practice (Public), social service providers	General Practice (Public)	Geriatrician
	Ng Teng Fong General Hospital	Geriatrician builds capability of primary care staff to assess and manage patients in primary care and the community setting	Age: 65 + CFS: 4–7	General Practice (Public), social service providers	General Practice (Public), social service providers	Geriatrician
SingHealth	Changi General Hospital	Geriatrician and community nurses support primary care clinicians to assess and manage patients in primary care setting	Age: 65 + CFS: 4–7	Hospital Emergency Department	General Practice (Private, Public)	Geriatrician
	Singapore General Hospital	Community nurses screen, assess and manage patients in the community, supported by family physicians	Age: 65 + CFS: 4–7	Community Nurse Posts, social service providers	Community Nurse Posts	Family Doctor and Nurse
	Sengkang General Hospital	Geriatrician and multi-disciplinary team support primary care clinicians to assess and manage patients in the primary care and community setting	Age: 65 + CFS: 4–7 Includes patients with dementia	General Practice (Private, Public), social service agencies, national senior care coordination agency	General Practice (Private, Public), community geriatrics nursing and rehabilitation facilities	Geriatrician

*CFS *Clinical Frailty Score

Although all five sites are designed and implemented differently, they share the common goal of providing comprehensive healthcare services to frail older adults in Singapore. Therefore, this evaluation seeks to holistically assess the pilot GSH models using a standardised process and outcomes framework while incorporating contextual information about the implementation and care experiences across the five sites. It is important for complex interventions to go beyond evaluating outcomes and create a response loop to provide qualitative insights for the future development and implementation of these care models.

## Study aims and hypotheses

Through this evaluation, we hope to gain a comprehensive understanding of the factors influencing the implementation approaches adopted by the GSH programmes and their outcomes. Our specific evaluation objectives are:

1. To assess the process of development and factors that influenced the implementation of the GSH programme;
2. To determine the influence of the GSH programme in a pre-post study to assess the health, quality of life, and user experience effects; and
3. To determine the influence of the GSH programme on healthcare utilisation and cost compared to matched controls.

 Our hypotheses are presented below. 

1. GSH sites offer a range of medical, social and other services through either direct provision or referrals. Given that the model is intended to bridge service gaps (conduct of CGA in the community), in the short term, we hypothesise an increase in the utilisation of appropriate services (rehabilitation, ambulatory services) in this time-limited programme.
2. GSH participants are expected to benefit from the comprehensive health and social services package and a multi-disciplinary team approach. With better care coordination and improved access, it is likely to elicit a higher level of satisfaction relative to comparator groups.
3. Education of the client about self-care and making decisions about potential care options with inputs from a multi-disciplinary team is expected to result in higher level of shared decision making and engagement relative to comparator groups.
4. Enrollment in GSH is hypothesised to result in better functional status and health outcomes, might reduce the healthcare utilisation (emergency hospitalisation, nursing home admission), caregiver burden and the associated indirect cost. In turn, we might expect overall costs to be lower compared to the comparison group.

The study will be reported according to established reporting guidelines for complex interventions using the evaluation segment of the Criteria for Reporting the Development and Evaluation of Complex Interventions in Healthcare: revised guidelines (CREDECI2; [35]).

## Methods

### Study design

The GSH is a complex intervention with multiple interacting components involving different organisational partners providing various types of care to frail older persons living in the community. We chose to adopt a mixed-methods approach, relying on the principle of complementarity, to achieve the specific study objectives mentioned above. Using mixed methods, the team can employ a qualitative approach to understand the circumstantial and programmatic factors that influence the implementation outcomes [36], complemented by quantitative approaches to measure the processes and outcomes stemming from the GSH.

The qualitative and quantitative components were collected concurrently, given equal weightage, analysed separately and integrated during the interpretation of the findings [37]. The Framework on Implementation Research developed for Client-Centred Medical Homes [38] is used as a sensitising framework to structure the work phases of this evaluation and the reporting of the results. In adopting this pragmatic approach, we acknowledge that there are subjective realities that can be objectively observed [39]. Quantitative research approaches are complemented with an in-depth exploration of contextual factors using a qualitative approach [37, 40]. Quantitative data also allows for the assessment of processes and multiple outcomes, such as health status and quality of life.

**Table 2:** Objectives and methods

Evaluation objectives	Aims	Methods
***Work package 1***		
1. To assess the process of development and implementation of the GSH programme	• To examine the perceptions, roles, responsibilities, and experiences in developing and implementing each programme • To understand the care model and workflow from the perspective of key partner organisations, for example, primary care providers, community and social service providers, for each programme • To explore differences in contextual factors across the five sites that have influenced implementation experiences and outcomes • To document observable key processes to support the achievement of desired outcomes	Qualitative: Semi-structured in-depth interview with key policy and programme decision-makers Qualitative: Semi-structured focus group discussions with health and social care professionals Qualitative: Participant observations Quantitative: Longitudinal monitoring of process indicators
***Work package 2***		
2. To determine the influence of the GSH programme in a pre-post study to assess the health, quality of life, and healthcare service experience	• To compare outcomes between baseline, 3- and 6-months for each programme	Quantitative: Pre-test post-test design using survey-based data collection
***Work package 3***		
3. To determine the influence of the GSH programme on healthcare utilisation and cost compared to matched controls.	• To assess the use of healthcare services and costs between participants and non-participants for each programme	Quantitative: Retrospective cohort design with propensity score matched comparators

All evaluation objectives will be studied for all sites except for Ng Teng Feng General Hospital (NTFGH), which will be evaluated based on Objective 1. The difference is based on the funded components of each site (Table 1). Other than NTFGH, the funding structure of the four sites includes the subsidy of patients’ healthcare costs. Meanwhile, NTFGH’s programme focuses on capability building, with the funding being utilised to cover the time spent by trainers and trainees but not the healthcare costs incurred by patients. Based on these considerations, evaluation objectives 2 and 3 would not be representative of the results of the programme.

The 4 section describes the work packages (a collation of data collection methods) that correspond to the evaluation objectives (Table 2). Each package is structured to describe the aim, study sample, procedure and data analysis to fulfil the objective.

### Work package for evaluation objective 1

#### Aims

Given the multi-agency and multi-professional setup of the GSH, it is crucial to consider the capability building process and implementation experiences across organisations and professional groups [41]. We aim to elicit the perspectives and experiences of professional stakeholders playing critical roles in the development and implementation of the programme through in-depth interviews [42] and focus group discussions (FGDs). Participant observations will be used to collect detailed information about each site’s culture and workflow. Finally, we use process indicators to describe the observable key processes that can be documented to support the achievement of desired outcomes.

#### Study sample/ data source

For semi-structured in-depth interviews, at least two participants representing each of the five implementation sites and the Ministry of Health will be identified through purposive sampling. They will include key policy and programme decision-makers who have the authority or have contributed significantly to the programme’s policy and implementation decisions. It will also include individuals familiar with the hospital’s overall frailty strategy. We aim to recruit an estimated number of 12 participants. The interviews are intended to examine the model conceptualisation, planning and implementation of the GSH at an early stage of this pilot study.

For FGDs, we plan for three rounds of such discussions with different stakeholders, namely the core implementation teams at the five sites, the partner organisations of the five sites, and the patients who have receive care from the GSH. The participants are identified through purposive sampling. In the first round of FGDs, at least five participants, comprising of members of the core implementation team will be identified at each of the five sites. They will include the healthcare professionals and administrative staff with time funded through the programme and those who have been providing services in the GSH for at least six months. We aim to recruit an estimated number of 25 participants. In the second round of FGDs, at least 5 participants, specifically the healthcare providers and administrative staff from partner organisations (e.g. primary care providers, community health and social service providers) will be identified at each of the five sites. Similarly, they are required to have provided services in the GSH for at least 6 months to take part in the study. We aim to recruit an estimated of 25 participants. Both the FGDs with core team members and partner organisations are intended to examine the perspectives and experiences in implementing the GSH and observe the process of collective sense-making [43]. In the third round of FGDs, we aim to identify at least ten participants who have received or are receiving care under the GSH for at least three months. To qualify for the study, the participants must be able to take part in the discussion for at least 60 min. For those who lack the capacity to do so, their primary caregiver is eligible for participation. The FGDs with patients are intended to understand their lived experience of receiving care from the GSH programme.

We will conduct participant observations to better understand the processes involved by shadowing three to five members of the staff over one week per site to account for day-to-day variations and job role differences. The observation will help us generate a more holistic understanding of the context and operations [43] and treatment processes (Fig. 1).

Finally, process indicators that measure the processes of care for all enrolled individuals will be collected and documented from the respective GSH sites to support the achievement of desired outcomes.

#### Study procedure

Written informed consent will be obtained from all participants. They will first be invited to complete a survey packet that consists of a basic demographic questionnaire and a scale to assess their experiences in the programme. Next, they will proceed to participate in the in-depth interview or FGD. The interview FGD guides were developed based on the key factors identified by Kodner and Kyriacou (2000) to be integral to the development and implementation of integrated care [44]. We chose this framework as it provided sufficient components for a holistic evaluation and a structure to assess similarities and differences in the operations of the different GSH sites.

Of the 15 factors identified by Kodner and Kyriacou (2000), the research team deliberated and identified 12 that were key to understanding the GSH implementation across the five sites. We renamed the factor “focus on continuum of care” as “patient-centred care” to reflect work done by providers to align with patients’ needs and preferences but at the same time to better differentiate this factor from “continuity of coverage and care” or having control over transitions between services and providers. A brief description of their relevance to the GSH is outlined in Table 3 below.

**Table 3:** Factors integral to understanding Geriatric Services Hub implementation

S/N	Factors	Brief description
1	Patient screening	Identifying frail older persons in the community for the GSH
2	Multi-disciplinary assessment	Conducting frailty assessment using a CGA, followed by multi-disciplinary team meetings involving geriatricians, geriatric nurses and allied healthcare professionals in the discussion of care.
3	Comprehensive service package	Developing and implementing individualised care plans, including referrals to frailty-related services to meet identified needs.
4	Network relationships	Partnerships and working arrangements between GSH site and partner organisations, such as primary care providers, community health and social service providers, including information sharing between them.
5	Care management	Planning care and coordinating care across time, place and discipline.
6	Continuity of coverage and care	Provider’s ability to help patients access frailty-related services across different settings and providers.
7	Seamless/Ease of transition	Patient’s ability to access frailty-related services and navigate between different settings and providers.
8	Teamwork	Roles and responsibilities of the GSH core team members; ongoing communication and collaboration among the multi-disciplinary group of providers.
9	Patient-centred care	The extent to which clinicians and patients work together to make decisions and select tests, treatments and care plans based on evidence that balances risks and intended outcomes with patient preferences and values.
10	Strategic planning	Stakeholder involvement in joint planning and community needs assessment
11	Funding mechanism	Structure of funding for health and social care.
12	System outcomes	Overall responsibility for the intended outcomes.

All interviews and FGDs will be audio-recorded and transcribed verbatim. Each interview is expected to last 90 to 120 min, whereas each FGD is expected to last 120 min. For the FGDs, we aim to balance homogeneity against the need for constructive tension by planning the group composition in such a way that reflects a similar frame of references based on participants’ roles and involvement in the programme, that is, the core implementation team, the partner organisations and the GSH patients [45].

For the participant observation, the research team will embed themselves in the day-to-day operations of the programme environment and take extensive field notes. Informal interviews will also be conducted to support our observations of the activities. The observational components serve to explore professional practices in service implementation, coordination, and collaborative interactions.

Quantitative process indicators will be developed based on the logic model with inputs from each of the five implementing sites to ensure they accurately describe the key processes that support the achievement of desired outcomes. Table 4 outlines the full list of requested process indicators.

**Table 4:** List and description of process indicators

Indicators	Measure	Definition	Data collection time-points
**Patient recruitment**			
Number of patients recruited by GSH sites	Receptivity towards GSH	Number of enrollees recruited into each GSH site after being referred	Monthly
Number, proportion of referred patients who fall within CFS 4–7	Accurate identification of frailty	CFS profiles of patients referred as scored by referral sources	Monthly
**Patient-focused care management**			
Number, proportion of CGA completed	Personalised care	Number of CGA completed vis-à-vis no. of assessments initiated	Monthly
Number, proportion of ICP developed	Personalised, goal-oriented care	Total no. of ICP developed vis-à-vis the no. of CGA completed	Monthly
**Coordination of care**			
Number of multi-disciplinary rounds/discussions	Team-based care	Number of multi-disciplinary team discussions conducted	Monthly
Number, proportion of referrals to services	Efficiency in care continuity	Number of referrals made to different services and the share of each service to the total no. of referrals	Monthly
Number, proportion of actualised referrals	Care continuity	Number of actualised first referrals at respective services vis-à-vis the no. of referrals made to each service^a^	Monthly
Appointment waiting time to first appointment	Efficiency in care continuity	Waiting time for a first appointment to a referred service	Monthly
**Capability building**			
Number of community-based staff trained to conduct specific activities (CGA, exercise)	Capability building	Number of community-based healthcare participants in training sessions organised by the GSH^b^	Quarterly

*CFS *Clinical Frailty Score, *CGA *Comprehensive Geriatric Assessments, *ICP *Individualised Care Plans, *GSH *Geriatric Services Hub

^a^Actualised first referrals refers to the number of first referrals where the patients the referrals were made for turned up

^b^Training sessions include preceptorship-based training and case discussions

### Data analysis

For the semi-structured in-depth interviews and focus group discussions, we will use the Framework Analysis approach [46] to generate important categories and themes that have influenced the development and implementation of the programme. Key steps outlined by Gale et al. (2013) will be adopted [47]. First, we will familiarise ourselves with the data by thoroughly reading the transcripts and listening back to the recorded interviews, if necessary. Field notes made during and after the interviews will be read alongside the transcripts to ensure that the context is taken into consideration. Based on the 12 factors adapted from Kodner and Kyriacou (2000) to describe the model and process of care at each of the five sites (Table 3), we will identify overarching categories where conceptually related codes will be grouped.

A preliminary list of codes will be derived from the literature [17, 29, 48–51] and our initial first impressions. Initially, three research team members will independently code the same two transcripts, allowing the codes to emerge inductively from the data in an open coding process. Subsequently, the team will compare and discuss the codes, agree on a set of codes, assign code labels, and provide each with a brief definition. This working analytical framework will be applied to subsequent transcripts using NVivo (Release 1.0) (released in March 2020). Once the data has been coded using the analytical framework, we will group codes that are conceptually similar under the identified categories in a matrix. We will refine and create new categories if necessary. Key themes will be generated from the codes by reviewing the matrix and making connections within and between categories. This process will be guided by the research objectives, the analytical framework and any new concepts generated inductively from the data. During the interpretation stage, we plan to take the analysis beyond describing the implementation at each site toward developing themes to offer possible explanations for what was happening across sites. The interpretation of findings will be discussed within the team and presented to selected respondents from the implementation sites for member checking.

The process indicators will be computed for each month and quarterly to monitor the programme’s progress. The relevant sub-populations accessing each care component will form the denominator for the computation of the indicators. Baseline characteristics will be described with mean and SD for continuous variables, and number and percentage for categorical variables.

#### Integration of qualitative and quantitative data

In the final interpretation of the data, we will bring together and triangulate the results from the various coordinated parts [52]. Findings from each component of a study will be listed to allow the evaluation team to look for convergence in findings, offer complementary information on the same issues and highlight discrepancies [53]. Results from interviews, FGDs and participant observations will be used to contextualise quantitative process indicators and for corroboration.

### Work package for evaluation objective 2

#### Aims

We will use a single cohort survey using a pre-test post-test design (pre-experimental design) to determine the influence of the GSH programme on patients’ health outcomes, their experience of care, and caregiver’s burden. There will be no randomised parallel control group due to programmes’ reluctance and anticipation of high refusal rates from patients. In addition, the GSH encompasses different care components tailored to each patient’s needs, which renders it impractical to conduct a ‘true’ experimental design with randomisation and a separate control group [54].

#### Study sample

We aim to recruit a target sample of 300 participants per GSH programme from the patients enrolled in the GSH. The sample was calculated based on the Barthel Index (100 points) – a scale to assess physical functioning, the key health outcome expected to improve due to participation in the GSH. We computed the sample size for each participating site using the dependent t-test to detect a small effect size of 0.2 (based on ß = 0.80, a = 0.05) [55]. The result was a minimum sample size of 156 per participating site. Allowing 20% rejection rate in the first instance and a subsequent attrition rate of 30%, we would need to approach 300 individuals in the first instance.

#### Study procedure

Interviewer-administered surveys will be conducted to collect data on demographic and programme outcomes (Table 5). After obtaining informed consent, participants will be asked to complete a survey estimated to take about 45 to 90 min up to three time-points – baseline (within 1 month of programme enrollment) and at 3-months and 6-months post-enrollment. The primary caregiver will also be asked to complete a 5 to 10-minute survey on caregiver burden at the same three time points. If a participant is clinically certified to have dementia, we will allow a proxy respondent to complete the survey on behalf of the main participant. Participants will receive a token of appreciation for their involvement in the study.

**Table 5:** List of indicators for measuring programme outcomes

Outcomes	Assessment	Measure
**Patient engagement**		
Shared decision making	collaboRATE For Patient – 5-point anchor scale	Patient’s experience of shared decision making
Patient activation	13-item Patient Activation Measure (PAM-13)	Level of patient activation, including ability to self-manage, maintain functioning, collaborate with healthcare providers, and access healthcare services
**Healthcare experiences**		
Experience of care delivered	Consumer Assessment of Healthcare Providers and System Clinician & Group Survey Version 3.0 (CG-CAHPS)	Patients’ experience with healthcare providers and staff in doctors’ offices
**Health status, adverse outcomes and quality of life**		
Functional status	Barthel Index of Activities of Daily Living (ADL)	Functional independence in ADL, such as feeding, bathing, and continence
Frequency of falls	Count of falls	Marker of poor health and declining function
Health-related quality of life	EuroOol-5D-5 L	Health-related quality of life in domains including mobility, self-care, usual activities, pain, anxiety and depression
	19-item Quality of Life Scale (CASP-19)	Quality of life in later life in domains including control, autonomy, self-realisation, and pleasure
**Caregiver burden**		
Level of caregiver burden	Zarit Burden Interview (ZBI)	Level of burden experienced by primary caregivers of older adults with dementia
**Direct and indirect cost**		
Societal cost	Client Service Receipt Inventory (CSRI)	Health, social and informal care use and cost

#### Data analysis

For survey-based collected outcomes, Generalised Linear Models (GLM) will be used to allow us to make inferences about the population when accounting for the within-subject correlation.

### Work package for evaluation objective 3

#### Aims

To determine the influence of the GSH programme on healthcare utilisation and cost within the current healthcare landscape, we will use a retrospective cohort design with propensity score-matched comparators. We opted for a quasi-experimental design due to a lack of a parallel control group.

#### Study sample

All programme enrollees are included and will be matched to a constructed comparator group using propensity score-matching. Both groups will be matched based on their probability of enrolling into the GSH conditional on baseline covariates [56].

#### Study procedure

To compare programme enrollees and the comparator group, we will establish an anonymised analytical dataset combined from three data sources. The first dataset will include the National Registration Identity Card (NRIC) number^1^ of all programme enrollees maintained by each of the five GSH sites. The NRIC for each enrollee will be assigned a unique identifier by a third party, which will subsequently be used to merge data across the datasets. The second dataset will include all the survey data collected by the evaluation team. The third dataset is maintained by the Ministry of Health. It will contain the use and system cost of healthcare services (primary care, specialist care, emergency services, inpatient care), as well as a nationwide survey on the health status of community-dwelling older adults from various walks of life. The comparator group would be derived from the third data set as it would confer us the highest chances of selecting comparators that were closest to the profile of programme enrollees.

#### Data analysis

For the retrospective cohort design, a propensity score conditional upon observed covariates will be computed for all cases. Variables will include sociodemographic information, disease burden measured by the Charlson Comorbidity Index [57], and physical functional status measured by the matching of CFS. We will match 2 comparators to 1 case using nearest-neighbour matching. In a Monte Carlo simulation, the mean squared error for a 2:1 match was minimised in 84% of the simulations compared with 68% for a 1:1 match [58]. Nearest neighbour matching based on a calliper of 0.01 of the standard deviation of the propensity score will be used [56, 59]. Only cases and comparators with propensity scores falling within a common support region range will be included in the analysis to ensure comparability of the two groups.

In the multivariable regression analysis of count data, Poisson distribution or Negative Binomial distribution (variance greater than the mean) will be used. Negative Binomial regression can be used for over-dispersed count data when the conditional variance exceeds the conditional mean. GLM will be used for modelling non-normally distributed continuous data such as length of stay and healthcare cost. The results will be presented as incidence-rate ratios (IRRs). All analyses will be performed using Stata/SE 16.1[60], with the level of significance set at 5%.

## Discussion

The GSH aims to identify and manage frailty in the community by providing comprehensive and coordinated care for frail older adults, and to achieve this through collaborative partnerships with various health and social service providers. The suite of services comprises of frailty identification, frailty assessment using a CGA, MDMs to discuss and develop individualised care plans, followed by referrals to frailty-related services and care coordination to facilitate the utilisation of these services. The five GSH pilot sites share these common programme features. However, due to differences in their innate organisational operations, the development of the GSH models was conceptualised differently by harnessing each respective site’s unique organisational strengths and leveraging on their existing relationships with partner organisations.

The motivation to conduct a mixed-methods evaluation is driven by the need to provide good quality evidence on the organisation of community-based frailty care. Integration between acute care and other forms of care in the community is becoming increasingly advocated [61]. As GSH is a model of care that aims to provide comprehensive care to frail older adults, it is essential to gain rich insights into the development and implementation of the programme. This understanding will help us determine each programme’s effects on patients’ health outcomes, programme cost-utilisation, and the programme replicability. Variations in the operations at each site demands that our evaluation methods be rigorous and consistent across all sites. This will enable us to identify essential links in each model and contribute more effectively to the future planning and implementation of care models that provide comprehensive care.

### Strengths and limitations

The study protocol has several strengths. Firstly, the mixed-methods approach allows in-depth understanding of the programme from the perspectives of different stakeholders, as well as the programme’s attributed health and cost outcomes. Secondly, this approach enables us to triangulate the findings, and cross-compare the validity and reliability of the findings through the different mediums of data collection. Finally, the choice of propensity score to match patients will allow a close match in baseline characteristics between treatment and comparator groups. This method attempts to replicate the balance in characteristics achieved by randomisation, thereby minimising selection bias. As a result, it contributes to the validity of conclusions to be made on healthcare utilisation and cost outcomes.

The protocol has several limitations that would affect implementation. Firstly, due to the dynamic nature of pilot programmes, adaptations will likely occur as implementation sites seek to refine their work processes to meet the evolving needs of frail older adults. However, our choice of a mixed-methods approach will provide insights into the context and changes over time and yet allow us to monitor a standard set of processes and outcomes over the pilot phase. Secondly, due to the lack of a shared Information Technology system between GSH sites and partner organisations, it will be challenging to track and monitor patients’ continued adherence to referred services. For some sites, information between partners will be shared via hardcopy documents. The collation of such information will require more manpower, which is not within the scope of this study. Thirdly, the study findings may not be generalisable to all frail older adults. The findings will be limited only to those who enroll into the GSH and are willing to participate in our study. Lastly, we are unable to include a parallel control group to ascertain the incremental effect of GSH across the entire range of outcomes. However, to address this limitation, we will deploy a propensity score-matching approach to derive the impact of GSH on healthcare utilisation and cost, which will support policymakers in their assessment of the programme’s financial sustainability.

In summary, the GSH is a multi-disciplinary care model that focuses on identifying and managing frailty in the community. This pilot programme is led by a core team of acute hospital-based healthcare professionals, in partnership with primary care providers, community health and social service providers, to increase access to essential services and ease transition across different services for frail older adults in the community. We believe that this evaluation study can provide invaluable insights into the process and outcomes of the GSH. Lessons learnt from this study will be disseminated to programme planners, implementers as well as policymakers and inform the design of similar programmes for frail older adults.

## Data Availability

Not applicable.

## Abbreviations

UK: United Kingdom
CGAs: Comprehensive Geriatric Assessments
ICPs: Individualised Care Plans
GSH: Geriatric Services Hub
RHS: Regional Healthcare Systems
CFS: Clinical Frailty Scale
GPs: General Practitioners
NTFGH: Ng Teng Fong General Hospital
FGD: Focus Group Discussion
PAM-13: 13-item Patient Activation Measure
CG-CAHPS: Consumer Assessment of Healthcare Providers and System Clinician & Group Survey Version 3.0
ADL: Activities of Daily Living
CASP-19: 19-item Quality of Life Scale
ZBI: Zarit Burden Interview
CSRI: Client Service Receipt Inventory
NRIC: National Registration Identity Card
GLM: Generalised Linear Models
IRRs: Incident-rate Ratios

## Footnotes

1 All persons residing lawfully in Singapore are issued a unique National Registration Identity Card (NRIC) number by the government.
